# Askin's Tumor in an Adult: Case Report and Findings on 18F-FDG PET/CT

**DOI:** 10.1155/2009/517329

**Published:** 2009-12-22

**Authors:** Gonca Kara Gedik, Oktay Sari, Tamer Altinok, Lema Tavli, Bugra Kaya, Pelin Ozcan Kara

**Affiliations:** ^1^Department of Nuclear Medicine, Selcuklu Medical Faculty, Selcuk University, 42075 Konya, Turkey; ^2^Department of Nuclear Medicine, Meram Medical Faculty, Selcuk University, 42080 Konya, Turkey; ^3^Department of Thoracic and Cardiovascular Surgery, Meram Medical Faculty, Selcuk University, 42080 Konya, Turkey; ^4^Department of Pathology, Meram Medical Faculty, Selcuk University, 42080 Konya, Turkey

## Abstract

Primitive neuroectodermal tumor (PNET) of the chest wall or Askin's tumor is a rare neoplasm of chest wall. It most often affects children and adolescents and is a very rare tumor in adults. In this case report, we present an Askin's tumor occurred in a 73-year-old male. The patient was admitted with a history of 3-month lower back pain and cough. In computed tomography, there was a lesion with dimensions of 70 × 40 × 65 mm in the superior segment of the lower lobe of the left lung. Positron emission tomography/computed tomography with 18F-flourodeoxyglucose revealed a pleural-based tumor in the left lung with a maximum standardized uptake value of 4.36. No distant or lymph node metastases were present. The patient had gone through surgery, and wedge resection of the superior segment of left lobe and partial resection of the ipsilateral ribs were performed. Pathology report with immunocytochemistry was consistent with PNET and the patient received chemotherapy after that.

## 1. Introduction

Primitive neuroectodermal tumors (PNETs) and Ewing Sarcoma are closely related malignant, small, round-cell tumors of soft tissues and bones [1]. Both of them strongly express the glycoprotein p30/32(CD99) encoded by MIC2 gene [1]. Recently, PNET and Ewing sarcoma have been categorized into a group known as the Ewing family of tumors because of their immunohistochemical, ultrastructural, and molecular similarities. 

PNETs are malignant tumors of the central nervous system and usually found in infants, children, and young adults. PNETs arise from the primitive nerve cells of the nervous system but they can also occur in outside the central nervous system (peripheral PNETs) including bone in the extremities, pelvis, and the chest wall. 

PNETs of the chest wall were originally reported by Askin et al. [2] in 1979 in 20 children and adolescents with a mean age of 14 years. Since that time, PNETs localized in thoracopulmonary region have been defined as Askin's tumors. 

Herein, we report a 73-year-old male patient with Askin's tumor and describe findings on dual-modality positron emission tomography/computed tomography (PET/CT) carried out with 18F-flourodeoxyglucose (18F-FDG). Askin's tumor is a disease of pediatric age group and few young adults have been reported [3, 4]. In older age group, we found only one reported case of Askin's tumor presented at age of 67 [5]. We report here one further case of Askin's tumor occurred in older age. 

## 2. Case Report

**Figure 1:**
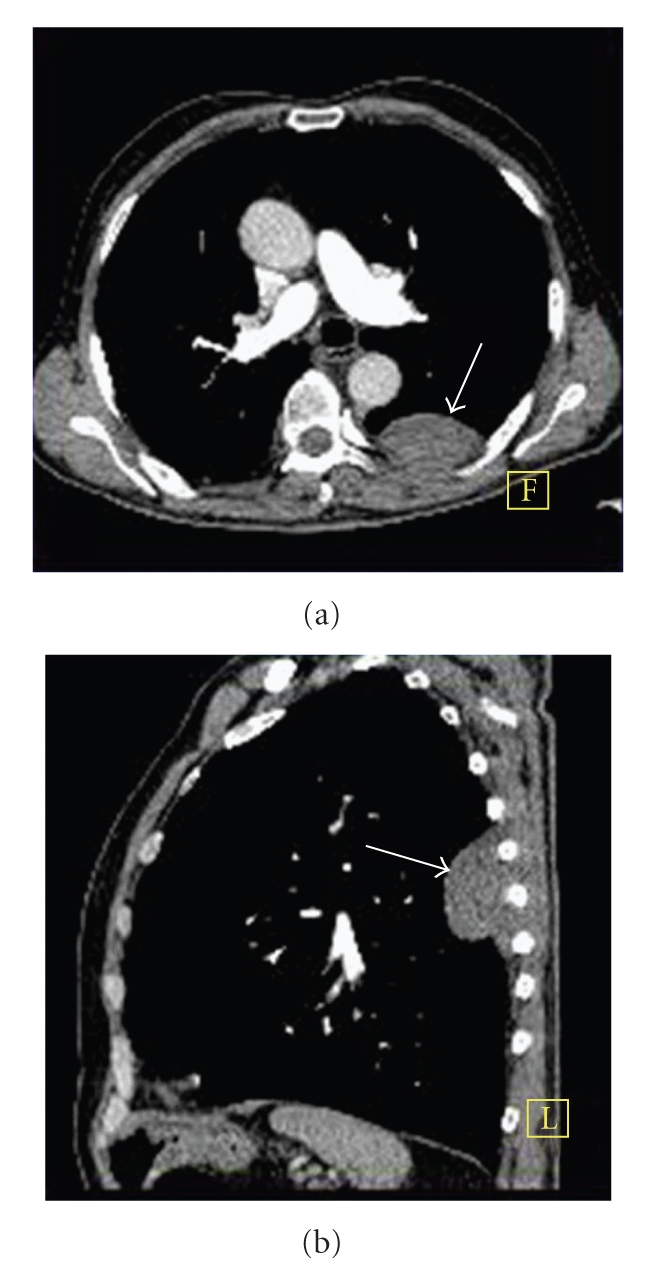
Axial (a) and sagittal (b) CT images demonstrate a lesion in the upper lobe of the left lung invading pleura and adjacent ribs (arrows).

A 73-year-old male patient without any history of smoking was admitted to hospital for lower back pain and cough on January 2009. From his history it was learnt that he was suffering from these symptoms for the last 3 months. Computed tomography confirmed the presence of a lesion with dimensions of 70 × 40 × 65 mm in the superior segment of the lower lobe of left lung. Invasion to the pleura and ipsilateral 6th, 7th, and 8th were also observed (Figures 1(a) and 1(b)). Fine-needle aspiration puncture performed with the guide of CT was suspicious for small cell carcinoma of the lung. Laboratory tests were in normal limits. 

The patient referred to nuclear medicine department for PET/CT. Dual-modality PET/CT imaging was performed after intravenous injection of 370 Mega Bequerel (10 mCi) 18F-FDG with a PET/CT scanner (Biograph, Siemens) from the top of the head to the middle of the thigh. Positron emission tomography attenuation correction was based on the CT data and coregistered attenuation corrected PET image and CT image were reviewed. 

**Figure 2:**
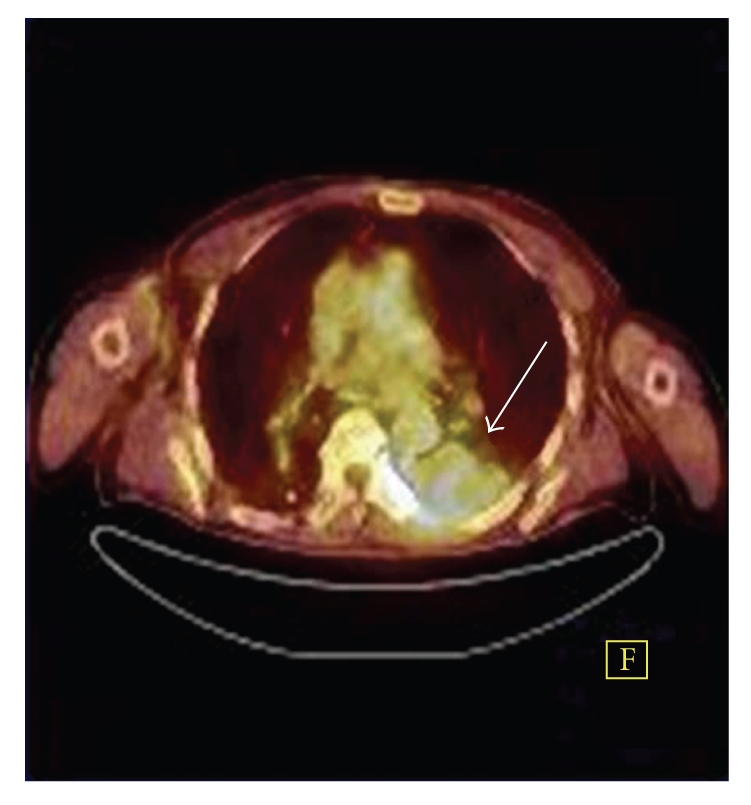
Axial-fused PET/CT image shows a pleural-based tumor with a calculated SUVmax of 4.36 (arrow).

PET/CT revealed a lesion in the superior segment of the lower lobe of left lung. Maximum calculated standardized uptake value (SUVmax) of the lesion was 4.36 (Figure 2). No additional findings including lymph node or distant metastases were noted in PET/CT. 

**Figure 3:**
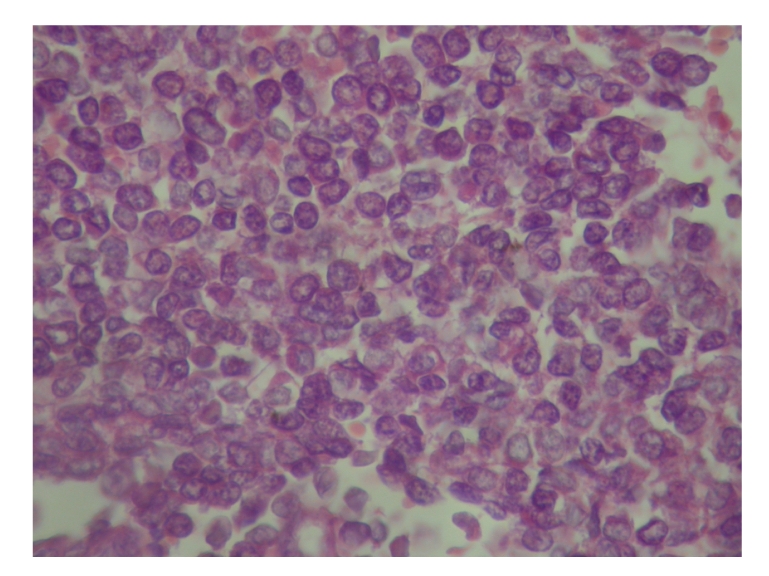
Small-sized round cells with scanty cytoplasm were seen in light microscopic evaluation (×40).

**Figure 4:**
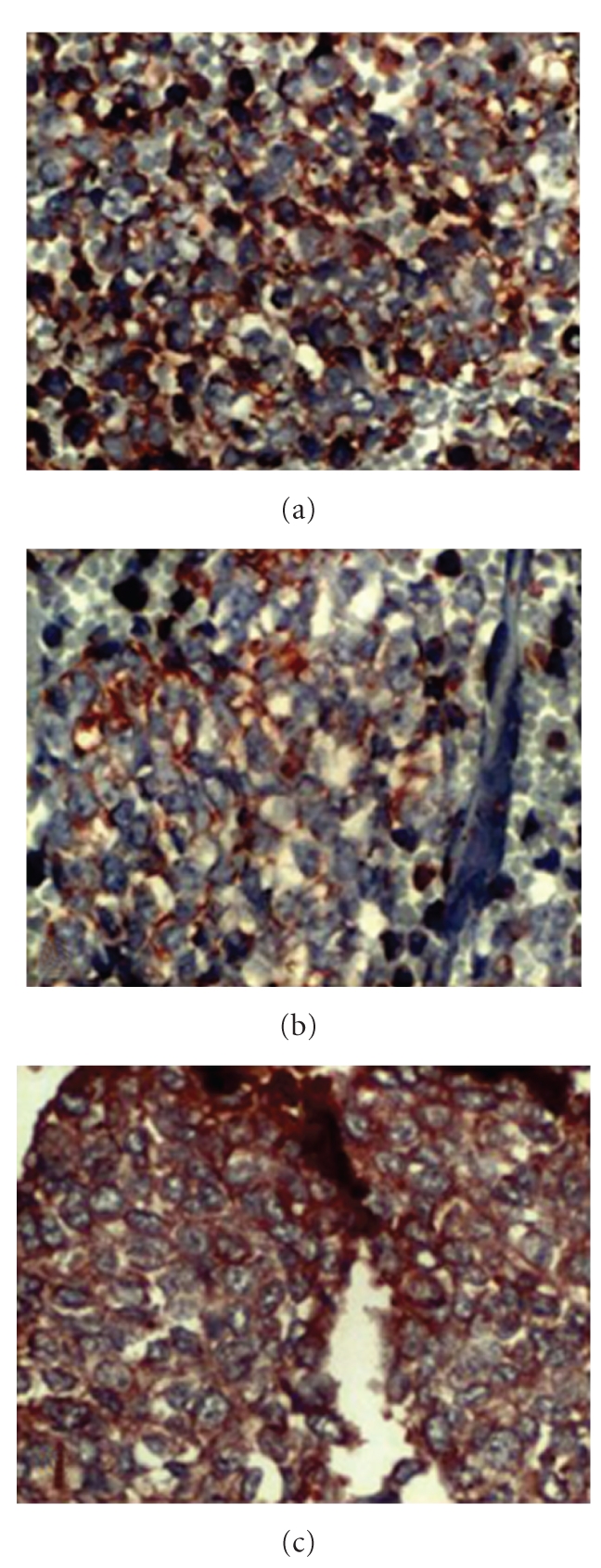
Immunohistochemistry revealed positivity with (a) CD 99, (b) vimentine, and (c) synaptophysin (×400).

Since no distant and lymph node metastases were present and the patient was operable, he had gone through surgery. During surgery, a lesion arising from the chest wall, infiltrating the adjacent ribs, was identified. Left thoracotomy, wedge resection of superior segment of the lower lobe of left lung, and partial resection of 6th, 7th, and 8th ribs were performed. Light microscopy showed small size round cells with scanty cytoplasm assembling in alveolar structures (Figure 3). After immunohistochemical analysis which showed positivity for CD 99, vimentine and synaptophysin (Figures 4(a), 4(b), and 4(c)), diagnosis of PNET was made. He is well and alive after 4 cycles of chemotherapy including adriamycine, cyclophosphamide, and vincryistine.

## 3. Discussion

PNET of chest wall is a distinct clinical and pathological entity arising from the soft tissue of the chest wall, occasionally from the rib cage and rarely from the periphery of the lung [6]. 

Radiologic features of central and peripheral PNETs have been documented [6, 7]. Soft tissue density and uncommon calcification were described on CT [6]. On magnetic resonance imaging (MRI), lesions were hyperintense compared to muscle on T1-weighted sequences and showed generally high and heterogenous signal on T2-weighted sequences [6]. Computed tomography was reported as valuable for evaluating tumor extension at diagnosis and response to chemotherapy [6]. Magnetic resonance imaging was found to be complementary to CT especially in the evaluation of extension of very large chest wall tumors [6]. 

In spite of the well-known radiologic features, limited data exists in the respect of the use of PET/CT fusion imaging in PNETs. Belonging to the same family, PET/CT has been shown to be useful for the detection of recurrence in Ewing sarcoma [8]. PET/CT was also reported to be useful in monitoring response to chemotherapy, radiotherapy, and postoperative evaluation of tumor resection sites in childhood sarcomas including Ewing sarcoma by By McCarville et al. [9]. 

Ewing family of tumors are high-grade malignancies which causes to expect high standardized uptake values (SUVs). However, reported SUVs are not high. Györke et al. [10] studied the impact of FDG PET for staging of Ewing sarcomas and PNETs and reported a mean SUV of 4.54 ± 2.79. Moreover, different SUVs were observed among primary tumors and metastases and this was attributed to different expression of glucose transporters in primaries and metastases. In that study, relatively low SUVs were also seen in lesions less than 15 mm and partial volume effect was discussed as a cause for low SUVs. 

In our patient, SUVmax of the lesion was calculated as 4.36 which was low like the other Ewing family of tumors and concordant with the findings of Györke et al. [10]. However, since the lowest dimension of the tumor of this patient was 40 mm, we thought that the expression of glucose transporters may be low in Ewing family of tumors compared to other high-grade malignancies and this may be an explanation for the low SUVs instead of partial volume effect. 

Except bronchoalveolar carcinomas, carcinoid tumors, and some subcentimeter-sized adenocarcinomas, FDG uptake is usually high in lung tumors. In a study by Tournoy et al., the role of 18F-FDG PET/CT in the staging of mediastinal lymph nodes was investigated and mean SUVmax of 8.34 was reported among all histologic subtypes of nonsmall cell carcinomas [11]. So besides bronchoalveolar carcinomas and carcinoids, PNETs of the thoracic wall should also be considered in the differential diagnosis of tumors in the thoracopulmonary region when low SUV is calculated in PET/CT. 

The present case is unusual for the age of presentation and demonstrating the 18F-FDG PET/CT findings. Reported adult patients with Askin's tumor are usually young adults. Our patient is older than the reported patients previously. We concluded that Askin's tumor must be kept in mind as one etiologic possibility in small round cell tumors of the thoracic wall not only in children and young adults but also in elderly. PET/CT may be helpful for characterization of the metabolic activity of the tumor and for investigation of distant metastases. 

## References

[1] Harimaya K, Oda Y, Matsuda S, Tanaka K, Chuman H, Iwamoto Y (2003). Primitive neuroectodermal tumor and extraskeletal Ewing sarcoma arising primarily around the spinal column: report of four cases and a review of the literature. *Spine*.

[2] Askin FB, Rosai J, Sibley RK, Dehner LP, McAlister WH (1979). Malignant small cell tumor of the thoracopulmonary region in childhood. A distinctive clinicopathological entity of uncertain histogenesis. *Cancer*.

[3] Cabezali R, Lozano R, Bustamante E (1994). Askin's tumor of the chest wall: a case report in an adult. *Journal of Thoracic and Cardiovascular Surgery*.

[4] Burge HJ, Novotny DB, Schiebler ML, Delany DJ, McCartney WH (1990). MRI of Askin's tumor. Case report at 1.5 T. *Chest*.

[5] Ravaux S, Bousqoet JC, Vancina S (1990). Askin's tumor in a 67-year-old man with cancer of the prostate. X-ray computed tomography aspects. *Journal de Radiologie*.

[6] Sallustio G, Pirronti T, Lasorella A, Natale L, Bray A, Marano P (1998). Diagnostic imaging of primitive neuroectodermal tumour of the chest wall (Askin tumour). *Pediatric Radiology*.

[7] Saifuddin A, Robertson RJ, Smith SH (1991). The radiology of Askin tumours. *Clinical Radiology*.

[8] Arush MW, Israel O, Postovsky S (2007). Positron emission tomography/computed tomography with ^18^fluoro-deoxyglucose in the detection of local recurrence and distant metastases of pediatric sarcoma. *Pediatric Blood and Cancer*.

[9] McCarville MB, Christie R, Daw NC, Spunt SL, Kaste SC (2005). PET/CT in the evaluation of childhood sarcomas. *American Journal of Roentgenology*.

[10] Györke T, Zajic T, Lange A (2006). Impact of FDG PET for staging of Ewing sarcomas and primitive neuroectodermal tumours. *Nuclear Medicine Communications*.

[11] Tournoy KG, Maddens S, Gosselin R, van Maele G, van Meerbeeck JP, Kelles A (2007). Integrated FDG-PET/CT does not make invasive staging of the intrathoracic lymph nodes in non-small cell lung cancer redundant: a prospective study. *Thorax*.

